# Importance of response to neoadjuvant chemotherapy in potentially curable colorectal cancer liver metastases

**DOI:** 10.1186/1471-2407-8-120

**Published:** 2008-04-25

**Authors:** Birgit Gruenberger, Werner Scheithauer, Robert Punzengruber, Christoph Zielinski, Dietmar Tamandl, Thomas Gruenberger

**Affiliations:** 1 Department of Medicine, Medical University Vienna, Vienna, Austria; 2 Department of Surgery, Medical University Vienna, Vienna, Austria; 3 Department of Internal Medicine, LKH Amstetten, Austria

## Abstract

**Background:**

Surgical resection of liver metastases arising from colorectal cancer is considered the only curative treatment option. However, many patients subsequently experience disease recurrence. We prospectively investigated whether neoadjuvant chemotherapy reduces the risk of recurrence following potentially curative liver resection. Special emphasis was directed to the importance of response.

**Methods:**

50 patients with resectable liver metastases received neoadjuvant XELOX or FOLFOX4 for six cycles (3 months). Complete resection of liver metastases was intended thereafter. Assessments included response rate, postoperative morbidity and recurrence-free survival.

**Results:**

An objective response was observed in 72% of all patients, including two complete responses. Chemotherapy was well tolerated and the majority of adverse events were mild to moderate (grade 1/2). Potentially curative R0 resection was performed in all patients and postoperative complications were observed in only 12%. The median recurrence-free survival was significantly influenced by tumor response with 24.7 months (95% CI: 4.50 to 44.97) in responding patients, 8.2 months (95% CI: 3.09 to 13.31) in patients with stable disease and 3.0 months (95% CI: 0 to 8.91) in patients with progressive disease.

**Conclusion:**

These data suggest that neoadjuvant Oxaliplatin based chemotherapy provides high response rates without increased risk of perioperative morbidity. Response to chemotherapy can lead to long-term recurrence-free survival. Neoadjuvant chemotherapy may identify best candidates for a potentially curative treatment approach.

## Background

Colorectal cancer is one of the most common malignancies in the Western world [1]. The liver is a frequent site of colorectal metastases, and 15% to 25% of patients have liver metastases at diagnosis [2,3]. In addition, 50% to 60% of patients with localized disease at diagnosis eventually develop advanced or metastatic disease. Median survival of patients with metastatic colorectal cancer treated with best supportive care alone is approximately 6 months [4]. The recent introduction of a number of new promising anticancer agents, like irinotecan or oxaliplatin in combination with 5-fluourocil, has resulted in response rates of 40% to 50% and a median survival of 15 to 20 months [5-7].

Despite advances in survival with chemotherapy, surgical resection of hepatic metastases is still considered the only curative option for patients with liver metastases and no extrahepatic disease, with 5-year survival rates after resection ranging from 25% to 45% [8,9]. However, even after a successful resection, the majority of patients will experience disease recurrence[10]. The role of dormant cancer cells, which subsequently will develop into metastases, has been established in experimental models [11]. The aim of systemic chemotherapy in patients with resectable liver metastases is to eliminate these tumor cells and thus reduce the risk of intrahepatic and extrahepatic recurrence. The majority of adjuvant studies in patients with colorectal cancer following liver resection, which used intra-arterial chemotherapy with or without systemic treatment, have failed to demonstrate a survival benefit [12-14].

Neoadjuvant chemotherapy appears promising, especially in patients with primarily resectable liver metastases with high risk of early recurrence, using doublet chemotherapeutic regimens containing oxaliplatin or irinotecan [15,16]. The intend of neoadjuvant chemotherapy is not only to reduce the risk of recurrence and downsize the liver metastases to enable less extensive surgery, but also to identify a group of patients who may benefit most from liver resection, namely the responders [17].

The aim of our prospective study was to determine the efficacy of neoadjuvant chemotherapy consisting of oxaliplatin plus 5-FU/LV or capecitabine in potentially curable patients with metastatic colorectal cancer and a high risk of tumor recurrence. Feasibility of this treatment approach has not been prospectively evaluated in terms of the prolongation of recurrence-free survival time in responding patients.

## Methods

### Patient selection

Patients were eligible for this prospective study if they had histologically confirmed technically resectable colorectal cancer liver metastases defined by a multidisciplinary team including liver surgeons, radiologists and medical oncologists, and at least one clinical risk factor for tumor recurrence defined by Fong *et al *[8]. The liver metastases had to be bidimensionally measurable, no limit in number and location of the metastases was given as long as they were defined resectable by the liver surgeon. In addition, eligible patients were required to have the following characteristics: age 19 to 80 years; World Health Organization (WHO) performance status of 0/1; adequate bone marrow reserve adequate renal and hepatic function.

Exclusion criteria were extrahepatic disease, prior palliative treatment, serious or uncontrolled concurrent medical illness and peripheral neuropathy (CTC > grade 1). They were not allowed to participate in any other clinical trial in the last 30 days. Written informed consent was required from all patients prior to study entry. The study was approved by the Institutional Review Board of the two participating centers.

### Treatment plan

Patients either received the standard FOLFOX4 regimen consisting of 85 mg/m^2 ^oxaliplatin (Eloxatin^®^; Sanofi-Aventis, Collegeville, USA) administered on day 1 as a 2-hours IV infusion; LV was given at the dose of 200 mg/m^2^as a 2-hour IV infusion, followed by 5-FU 400 mg/m^2 ^as IV bolus, and then, 600 mg/m^2 ^as a 22-hour continuous IV infusion, on days 1 and 2 or a XELOX regimen consisting of oxaliplatin 130 mg/m^2 ^administered as a 3-hour infusion on day 1 plus capecitabine (Xeloda^®^) 2,000 mg/m^2^/d po days 1 to 7 of a 2 week cycle.

Treatment courses were repeated every 2 weeks for a total of six courses unless there was prior evidence of progressive disease. Follow-up examinations including CT of the chest and abdomen and tumor marker measurement were carried out every 3 months during the first 2 years, every 6 months for the following 3 years and once yearly thereafter.

Allocation to FOLFOX4 or XELOX was based on institutional preference of two oncology departments because FOLFOX and XELOX were considered equally as first line treatment of colorectal cancer.

### Toxicity and dose modification guidelines

Adverse reactions were evaluated according to the National Cancer Institute Common Toxicity Criteria (NCI-CTC, Version 2.0) [18]. If patients experienced a grade 4 hematological or ≥ grade 3 non-hematological adverse event, the dose of chemotherapeutic drugs was reduced by 25% for all subsequent doses. In addition, for persistent severe neurotoxicity, despite a 25% dose reduction, oxaliplatin was temporarily withdrawn, with maintenance of 5-FU/LV or capecitabine, until recovery. Treatment was delayed for up to 2 weeks if the absolute neutrophil count was <1,500/μL or the platelet count was <100,000/μL. Subcutaneous erythropoietin was recommended for patients with hemoglobin <10 g/dL. Patients who required more than 2 weeks recovery from an adverse reaction (> grad 1) were excluded from this protocol.

### Pretreatment evaluations and assessment of response

Prior to therapy, all patients were assessed by physical examination, routine hematology and biochemistry analyses, and CT-scans of the thorax and abdomen to define the extent of disease. Following the initial assessment, complete blood cell counts and serum biochemistry analyses were obtained at least once every course of treatment. Carcinoembryonic antigen (CEA) levels were assessed every 4 weeks. Subjective symptoms, physical examination results, performance status and all adverse reactions were recorded before each treatment cycle according to the CTC criteria [18]. Tumor size was measured after six cycles by CT scan or MRI, and response rate was evaluated according to RECIST criteria [19].

### Statistical Analyses

The primary end point was response rate according to RECIST criteria; secondary endpoints were resectability rate and perioperative morbidity and mortality. The prolongation of overall and recurrence-free survival in responding patients were additional secondary endpoints.

The study design to predict the number of patients necessary for statistical validity (2-sided) was based on the assumption that treatment with XELOX or FOLFOX increases recurrence free survival time of responders compared to patients who progressed on therapy by 10 months. Alpha was set at 0.05, beta at 0.2, yielding a power of 80%; The calculated sample size for an acquisition period of 24 months and a follow up period of at least 30 months was 38 patients.

The Kaplan-Meier method was used to estimate the median overall survival and recurrence-free survival, applying Log-Rang comparison. Uni- and multivariate analyses and survival figures were plotted using SPSS for Windows version 11.5.

### Surgical technique

A CT-scan of the thorax and abdomen was performed at the end of the sixth cycle of chemotherapy to exclude extrahepatic disease. In addition, an echocardiogram, an ECG, indocyanine green clearance and a lung function test were performed routinely preoperatively. An intraoperative ultrasonography was carried out to confirm the number and size of metastases, to determine their relationship with vascular and biliary structures and for exclusion of further intrahepatic metastases. Liver resection was performed 2 to 5 weeks after the last administration of chemotherapy, and all patients must have recovered from any severe side effects of chemotherapy. Curative liver resection was obligatory, which included the resection of all liver metastases with a negative margin in a single procedure.

## Results

Between May 2001 and November 2003, 50 patients with colorectal cancer and resectable liver metastases were included into this prospective, non-randomized trial. Selected baseline demographics and disease characteristics are shown in Table 1. The enrolled patients had a median age of 62 (range 36 to77) years; metastases were synchronous in 35 patients (70%) and metachronous (diagnosed at least 6 months after the primary tumor) in 15 patients (30%). All patients received neoadjuvant chemotherapy for 3 months with 30 (60%) and 20 (40%) patients receiving XELOX and FOLFOX4, respectively. Patient characteristics were similar between both groups.

**Table 1:** Patients characteristics

**Parameter**	**All patients (n = 50)**
Age, median (range)	62 (36 to 77)
Sex (male/female)	34/16
ECOG performance status, n (0/1)	49/1
Disease stage, n (%)	
M0/M1	15 (30)/35(70)
N0/N1,2	16 (32)/34 (68)
G1/2/3	7 (15)/41(81)/2 (4)
Number of metastatic lesions (%)	
1	15 (30)
2–3	11 (22)
≥ 4	24 (48)
Lymph-node positive primary tumors, n (%)	34 (68)
Duration of metastatic disease < 12 months, n (%)	37 (74)
CEA > 20 ng/mL, n (%)	15 (30)
Synchronous metastases, n (%)	35 (70)

ECOG = Eastern Cooperative Oncology Group; CEA = carcinoembryonic antigen

### Toxicity

Hematological toxicity was observed in 27 (54%) patients, which included one (2%) patient with grade 3 neutropenia and one (2%) patient with grade 4 thrombocytopenia; all other hematological toxicities were mild to moderate (grade 1 or 2). There were no episodes of febrile neutropenia or bleeding. Non-hematological side effects were reported in 33 (66%) patients (Table 2). The most common mild to moderate (grade 1 or 2) non-hematological side effects were nausea (n = 17; 34%), diarrhea (n = 6;12%), vomiting (n = 3; 6%), fatigue (n = 4; 8%), peripheral neuropathy (n = 22; 44%) and hand-foot syndrome (n = 9; 18%). Grade 3 non-hematological side effects were observed in 5 (10%) patients, including diarrhea (n = 2; 4%), peripheral neuropathy (n = 2; 4%) and vomiting (n = 1; 2%). No grade 4 non-hematological side effects were observed.

**Table 2:** Selected adverse events

**Adverse event**	**Incidence (%)**		
	**Grade 1/2**	**Grade 3**	**Grade 4**

Neutropenia	24	2	-
Anemia	46	-	-
Thrombocytopenia	38	-	2
Nausea	34	0	-
Diarrhea	12	4	-
Vomiting	6	2	-
Fatigue	8	-	-
Peripheral neuropathy	44	4	-
Hand-foot syndrome	18	-	-

The initial dose of capecitabine was reduced (25%) in two patients receiving XELOX due to grade 3 diarrhea. A 25% dose reduction of oxaliplatin was performed in two patients, one each receiving XELOX and FOLFOX4, due to grade 3 peripheral neuropathy. A dose reduction of both agents was performed in two patients due to grade 4 thrombocytopenia (one XELOX patient) and grade 3 vomiting (one FOLFOX4 patient).

### Response to chemotherapy

Tumor response was evaluated in all 50 patients. Only two patients failed to receive all six cycles of chemotherapy (one patient experienced progressive disease after three cycles and the other patient discontinued after four cycles due to personal reasons). An objective response was observed in 36 (72%) patients (Table 3). Two (4%) patients were reported radiologically to have a complete response (CR); one was pathologically confirmed and viable tumor cells were discovered during pathology in the other patient; 34 (68%) patients had radiological confirmed partial response (PR). An additional 10 patients (20%) demonstrated with stable disease (SD) and only four patients (8%) had disease progression (PD). A reduction in CEA levels was observed in 63% of the patients, whilst levels were unchanged in a further 19% of patients. Tumor response was similar in patients who received XELOX or FOLFOX4, respectively (complete/partial response, 77% versus 65%; stable disease 13% versus 30%; progressive disease, 10% versus 5%).

**Table 3:** Analysis of efficacy

**Efficacy measure**	**All patients (n = 50)**
Objective response, n (%)	36 (72)
Complete response	2 (4)
Partial response	34 (68)
Stable disease, n (%)	10 (20)
Progressive disease, n (%)	4 (8)
Mean overall survival, months (95% CI)	38.0 (32.65 to 43.35)
Median recurrence-free survival, responders; mts (95% CI)	24.73 (4.50 to 44.97)

### Liver surgery, pathology, morbidity and mortality

R0 resection was performed in all 50 patients. Unisegmental resections were performed in 10 (20%) patients, bisegmentectomies were necessary in 13 (26%) patients and 27 (54%) patients underwent major hepatectomies (≥ 3 segments). Perioperative blood transfusions were given to 16 (32%) patients, but only seven patients required more than two units of packed red cells.

Postoperative complications were observed in only six (12%) patients; two patients each experienced wound healing problems, bilioma and an abscess formation. There was no 60 day mortality and median duration of hospitalization was 9 (range 5 to 29) days.

### Recurrence-free and overall survival

**Figure 1:**
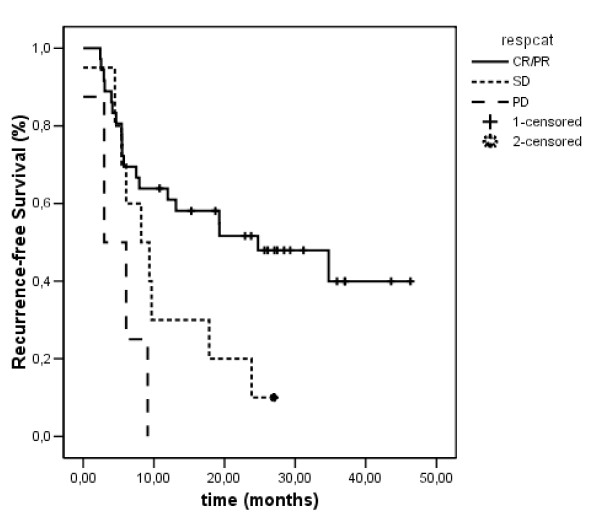
**Recurrence-free survival estimates**; PR = partial response, SD = stable disease, PD = progressive disease.

The median duration of post-operative follow-up is 31 months (95% CI: 27.28 to 35.05). Recurrence of disease was observed in 32 (64%) patients. The site of recurrence was intrahepatic in 16 patients, extrahepatic in 10 patients and both intra- and extrahepatic in 6 patients. There were no marginal recurrences. The median duration of recurrence-free survival was 12.0 months. The duration of median recurrence-free survival significantly differed according to tumor response following neoadjuvant chemotherapy, with 24.7 months (95% CI: 4.50 to 44.97) in responding patients, 8.2 months (95% CI: 3.09 to 13.31) in patients with stable disease and 3.0 months (95% CI: 0 to 8.91) in patients with progressive disease (p < 0.004), Figure 1. Palliative chemotherapy was administered to almost all patients with recurrent disease in the different response categories (84% in PR/CR patients, 88% in SD pts and 100% in PD pts), surgery for recurrence was performed in one patient in each response group and palliative care was given to two patients with recurrence in the initially responding group.

The median overall survival has neither been reached in responding patients nor in the entire population. In contrast, median overall survival in patients with stable disease was significantly longer than patients with progressive disease (21.2 months [95% CI: 7.65 to 34.82] vs 12.1 months [95% CI: 3.61 to 20.53]; p = 0.046). Mean overall survival was 38 months (95% CI: 32.65 to 43.35).

Multivariate analyses revealed response to neoadjuvant chemotherapy highly significant correlated to improved recurrence-free survival (p = 0.002) and overall survival (p = 0.017). Other factors in the RFS model were number of metastases (p = 0.040), synchronous vs metachronous metastases (p = 0.395) and lymph node status of the primary CRC (p = 0.243). All other factors in the model for OS were negative: number of metastases (p = 0.732), time of metastases (p = 0.642) and lymph nodes (p = 0.064).

## Discussion

To date, resection of liver metastases in patients with colorectal cancer is the only curative treatment option [8,9]. However, the majority of patients develop recurrence following resection, either intra- or extrahepatically, within 18 months after resection [20]. This may suggest that many patients do not optimally benefit from surgery and underlines that new treatment strategies are necessary. Especially patients with multiple known risk factors for early recurrence do certainly not benefit from a sole surgical approach. According to Fong's risk factor definition the majority of the patients in our trial would qualify for an overall survival of less than 30% after 5 years [8].

Available data, the published majority of which are from retrospective studies, indicate that neoadjuvant chemotherapy is appropriate for the treatment of colorectal cancer liver metastases [21,22]. Neoadjuvant chemotherapy has a number of potential benefits in patients with resectable liver metastases. These benefits include the potential for improved selection of patients for resection by excluding patients who progress during neoadjuvant therapy, in whom from our current knowledge surgery would be inappropriate. Additionally, the response or tolerability to a neoadjuvant chemotherapy regimen will help determine the optimal adjuvant chemotherapy. The beneficial effect of the perioperative chemotherapy setting was recently supported by the results of the EORTC 40983 study presented at the 2007 Annual Meeting of the American Association of Clinical Oncology [23]. Although a high percentage of patients with a low recurrence risk profile were randomized in this trial (e.g. over 50% pts with a single liver metastasis) the analyses showed a significant 3 year recurrence-free survival benefit in eligible patients.

An objective response rate of 72%, including one patient with complete pathological response, was observed in our study. The decrease of tumor volume reduces the magnitude of liver resection whilst sparing normal hepatic tissue and improves the complete resection rate. A significant tumor response may also allow for easier resection and improved postoperative recovery. In addition to the 72% of patients who responded to neoadjuvant chemotherapy, a further 20% of patients had stable disease. Even those who progressed during neoadjuvant chemotherapy did not become unresectable, however the usefulness of performing liver resection in progressive patients is not supported by our results, because all these patients relapse within 9 months after a potential curative approach.

In our study, a median recurrence-free survival of 12.0 months was observed with neoadjuvant XELOX or FOLFOX4. Although patients have been followed for 31 months, median overall survival for all and responding patients has not been reached; this is in contrast to those who were stable or progressed under neoadjuvant chemotherapy. Interestingly, these findings may indicate that a response to neoadjuvant chemotherapy may identify patients with high long-term survival rates and those appropriate for liver resection, and therefore potentially curable. The significant difference identified for recurrence-free and overall survival if response to chemotherapy occurs does support the value of neoadjuvant therapy even in resectable patients.

A previous study of patients who received neoadjuvant chemotherapy and surgery indicated that patients with progressive disease had significantly worse 5-year survival (8%) compared to patients with stable disease (30%) or responsive disease (37%; p = 0.0001) [24]. Recurrence-free survival and overall survival data from our study support these previous findings and may suggest that resection is appropriate only in responding and probably in stable patients. These results add to the current discussion of evaluating 2^nd ^line treatment in progressive patients prior to an optional surgical approach [25].

In an attempt to predict the survival of patients undergoing liver resection, multivariate analyses have identified factors predictive of recurrence or poor prognosis, including primary disease stage, number and volume of metastases and preoperative CEA concentration [8]. Neoadjuvant chemotherapy has at least in our study improve these unfavorable prognostic factors in over two third of the patients, and may therefore be considered an ideal parameter to estimate prognosis in patients diagnosed with metastatic colorectal cancer. Response to treatment positively influenced suspected worse prognosis (according to a high Fong score) in our study group (data not presented).

Phase II and III clinical trials have demonstrated that the addition of bevacizumab to standard chemotherapy regimens significantly improves response rates, overall survival and progression-free survival compared with chemotherapy alone [26-28]. In addition, cetuximab (Erbitux^®^) has demonstrated good response rates in previously treated patients with metastatic colorectal cancer and recently proofed effective in the first line setting [29-31]. Response rate is an important surrogate for neoadjuvant therapy. Therefore, the encouraging response rates produced with biological agents in combination with chemotherapy suggest the potential for optimization of neoadjuvant therapy. Studies evaluating the efficacy of neoadjuvant therapy consisting of biological therapy are ongoing and preliminary results are encouraging [32].

Advances in surgery including precise preoperative imaging and control of intraoperative bleeding have improved outcomes in the resection of colorectal liver metastases [17]. A low rate of perioperative and postoperative complications were observed in the present study. Similarly, no cases of operative mortality were reported. These findings are consistent with previous studies, indicating that liver resection after neoadjuvant chemotherapy is feasible without increased morbidity and mortality risk, particularly in experienced hands [16,23,33].

## Conclusion

We have shown that neoadjuvant chemotherapy in potentially curable patients with metastatic colorectal cancer and high risk of tumor recurrence using neoadjuvant XELOX or FOLFOX4 provides high response rates that allowed complete resection in all patients. The major issue in the attempt to cure metastatic colorectal cancer, the prolongation of recurrence-free survival was demonstrated in responding patients in our study. In addition, the chemotherapy regimens were well tolerated and low rates of complications, morbidity and mortality were observed. Although further studies are required to clarify its potential role, neoadjuvant chemotherapy with XELOX or FOLFOX4 should be considered as a valid treatment option in patients with resectable colorectal cancer liver metastases.

## Competing interests

The authors declare that they have no competing interests.

## Authors' contributions

BG designed the study, performed the statistical analyses and wrote the manuscript. WS, CZ and RP participated in its design and helped to draft the manuscript. DT did the data collection and analyzes. TG did the study conception and performed the interpretation of the data. All authors read and approved the final manuscript.

## Pre-publication history

The pre-publication history for this paper can be accessed here:


